# Prenatal diagnosis of persistent right umbilical vein – Incidence and clinical impact. A prospective study

**DOI:** 10.1111/ajo.12791

**Published:** 2018-03-02

**Authors:** Arkadiusz Krzyżanowski, Dariusz Swatowski, Tomasz Gęca, Maciej Kwiatek, Aleksandra Stupak, Sławomir Woźniak, Anna Kwaśniewska

**Affiliations:** ^1^ Department of Obstetrics and Pathology of Pregnancy Medical University of Lublin Lublin Poland; ^2^ Department of Gynecology Medical University of Lublin Lublin Poland

**Keywords:** anomalous venous system, fetal malformations, persistent right umbilical vein, prenatal diagnosis, ultrasounds

## Abstract

**Background:**

Persistent right umbilical vein (PRUV) is usually an isolated finding but it may be accompanied by other fetal malformations.

**Aims:**

We aimed to determine the incidence of prenatally diagnosed PRUV in a referral population, assess the neonatal outcome and discuss the findings together with those from previous publications.

**Materials and methods:**

A total of 2360 women with low‐risk singleton pregnancies were examined in the second and third trimesters. A transabdominal convex volume transducer was used. B‐mode was applied in each patient. Scanning of the venous system included imaging of the target vessels with two‐dimensional colour Doppler mapping. The diagnosis of PRUV was made in a transverse section of the fetal abdomen. Three‐dimensional ultrasounds were performed as necessary, when anomalous cases were encountered.

**Results:**

The incidence of PRUV in our population was 12/2360 = 0.5%, and it was higher than in other retrospective studies. In 75% (*n *=* *9), PRUV was an isolated finding where delivery was uneventful and the postnatal outcome was favourable. In two cases PRUV was accompanied by omphalocele, and in one case by tetralogy of Fallot and single umbilical artery.

**Conclusions:**

PRUV is an uncommon prenatal finding. Screening for this anomaly can be easily performed in all pregnant patients. A diagnosis of PRUV should be followed by a thorough fetal morphology scan in order to exclude any other malformations, especially those of the cardiovascular system.

## Introduction

The anomalies of the umbilical veins may involve the persistence of embryological structures, abnormal insertion, course and supernumerary vessels. The majority of the anomalies of the venous system appear infrequently, and some of them may be completely asymptomatic. In early development, both umbilical veins (UVs) are connected to the sinus venosus. Obliteration of the right UV begins at four weeks gestation and at seven weeks it disappears.^1^, ^2^ The left one, which is connected to the left portal vein (PV) in the fetal liver, then transports all the blood. When the right UV remains open it carries oxygenated blood to the heart. It may coexist with the left UV as an intrahepatic supernumerary structure.^3^

Persistent right umbilical vein (PRUV) is an altered embryonic development, in which the left umbilical vein regresses and the right vein remains open. The precise incidence of this lesion has never been established; however, recent studies have demonstrated that PRUV is more common than previously thought, and occurs in 1/250–1/1250 pregnancies.^4^, ^5^, ^6^ The exact incidence of PRUV might be higher, but the abnormality can be easily overlooked during standard ultrasound for abdominal circumference measurement. Better ultrasound techniques and colour Doppler and three‐dimensional ultrasounds (3D US) may help in the diagnosis.^7^, ^8^, ^9^ The cause of PRUV remains unknown. Thrombus obstruction, teratogens or folic acid deficiency are possible etiologies.^5^, ^10^, ^11^ More recent retrospective studies have proven that fetuses with isolated PRUV (76.3–98.6%) have a good prognosis.^4^, ^12^, ^13^ However, PRUV may be associated with the congenital absence of the ductus venosus (DV) and other severe fetal malformations.^7^, ^10^, ^14^ In isolated cases when the DV is normally connected and the portal system has all its branches, the haemodynamics are supposed to be normal. However, a close follow‐up is necessary to detect early signs of haemodynamic decompensation in the absence of DV.^4^, ^15^

The purpose of this prospective study was to determine the incidence of prenatally diagnosed PRUV in a referral population, assess the neonatal outcome and discuss the findings together with those from previous publications.

According to the available databases of worldwide literature, this is the first prospective study in Europe and only the fourth worldwide.^4^, ^16^, ^17^

## Materials and Methods

**Figure 1:**
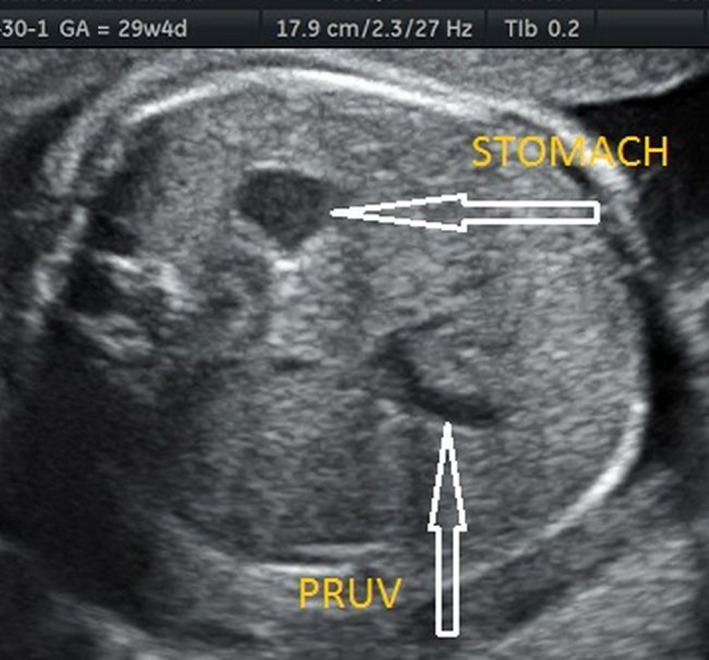
Persistent right umbilical vein (PRUV) visualised in a cross‐section of the fetal abdomen (B‐mode). PRUV tracks toward the stomach, to the left.

**Figure 2:**
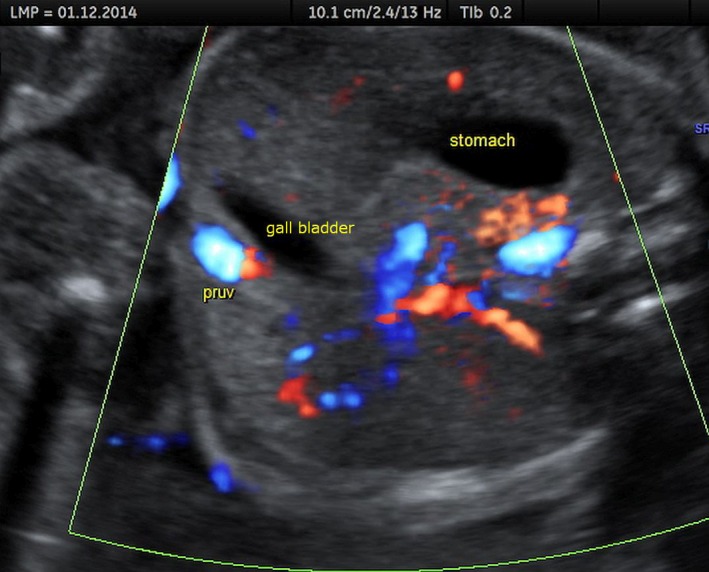
Persistent right umbilical vein (PRUV) visualised in a cross‐section of the fetal abdomen (colour Doppler). Gallbladder is situated medial to the UV.

**Figure 3:**
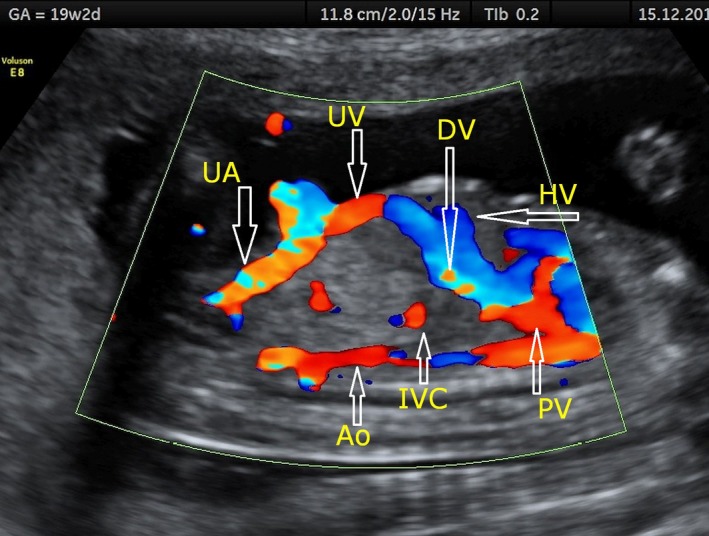
Ductus venosus visualised in a longitudinal plane. DV, ductus venosus; UV, umbilical vein; UA, umbilical artery; HV, hepatic vein; PV, portal vein; IVC, inferior vena cava; Ao, aorta.

**Figure 4:**
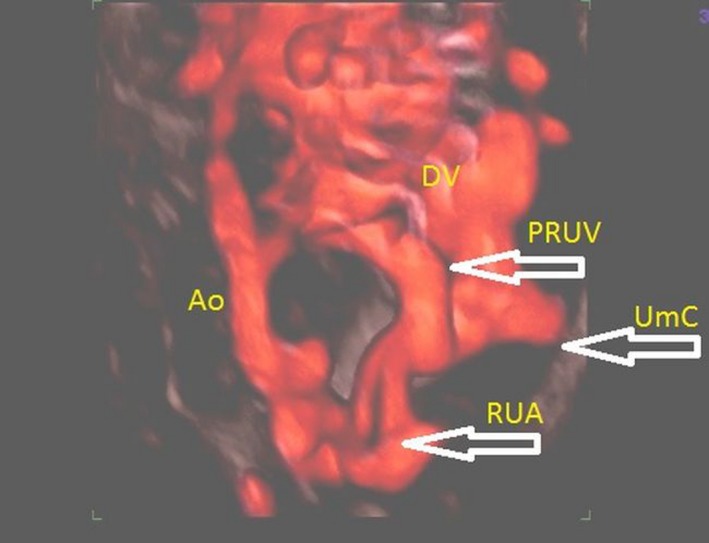
Persistent right umbilical vein (PRUV) in 3D power Doppler imaging of a fetal venous system. DV, ductus venosus; PRUV, persistent right umbilical vein; RUA, right umbilical artery; UmC, umbilical cord; Ao, aorta.

Women with low‐risk singleton pregnancies presenting for targeted organ scanning in the second and third trimesters were examined between January 2012 and March 2016 in a tertiary care centre in Lublin, Poland. The gestational age was established from the date of the last menstrual period and the first trimester US. A detailed anatomical evaluation of the fetuses was performed by two sonographers both certified by both the Polish Gynecological Society (PTG) and the Fetal Medical Foundation (FMF). GE Voluson E8 US machine (GE Medical Systems, Milwaukee, WI, USA) equipped with a transabdominal convex volume transducer with insonation frequencies of 4–8 MHz was used. B‐mode was applied in each patient. Scanning of the venous system included the imaging of target vessels with two‐dimensional colour Doppler mapping. The diagnosis of PRUV was conducted in a transverse section of the fetal abdomen. The sonographic criteria included: (i) an aberrant course of the PV toward the stomach; (ii) the fetal gallbladder being medial to the UV; or (iii) the connection of the UV to the portal vessels curving toward the stomach (Figs 1 and 2). The presence of the DV was confirmed in a typical longitudinal plane showing UV, left hepatic vein, right portal vein, DV, inferior vena cava (IVC) and pulmonary vein (Fig. 3). Fetuses with situs inversus, situs ambiguous and heterotaxy (left and right isomerism) were not involved. 3D US were performed as necessary when anomalous cases were encountered (Fig. 4). The imaging of the umbilical vein was similar to that described in other studies.^14^, ^15^ During the 3D data acquisition the women were asked to hold their breath. A medium wall filter and a gain of 50% were used. The volume angle was set at 55°.

In the cases of PRUV the diagnosis was confirmed by the second sonographer. Detailed fetal US and echocardiography were then performed to detect any other anomalies. In all cases of detected anomalies, a fetal karyotyping was offered. After birth, the infants were evaluated by paediatricians for any additional abnormalities. The prenatal sonograms and neonatal outcome data of the affected individuals were reviewed. Our findings were analysed together with the findings retrieved from the scientific literature.

The protocol was approved by the Institutional Review Board. In Poland, no written permission is needed to use or evaluate any data obtained during diagnostic examinations or standard therapeutic procedures for any scientific analysis. Any data provided the patients' name, religion, sexuality, ethnicity and so on, are not given in the final report.

## Results

There were 2360 women included in the study group. Their demographic characteristics are summarised in Table 1.

**Table 1:** Obstetric background characteristics of the study group of 2360 low‐risk women

Parameter	Range	Mean	SD
Maternal age (years)	16–48	31.5	4.71
GA at diagnosis (weeks)	15 + 0 to 41 + 0	25 + 0	5.91
Gravidity	1–9	1.9	1.06

PRUV was diagnosed in 12 women (0.5%). The median gestational age at diagnosis was 21 + 6 ± 2.5 weeks. The median maternal age in PRUV cases was 31 ± 4.39 years. In all cases, an intrahepatic type of PRUV (see discussion here) with present DV was observed. In nine cases (75%), PRUV was an isolated finding. In this group, seven healthy babies were delivered at term, and teo prematurely between 35–37 gestational weeks. The delivery was uneventful and the postnatal outcome was favourable in all cases of isolated PRUV (Table 2). In one fetus with normal karyotype, PRUV was accompanied by omphalocele. In another case of PRUV coexisting with omphalocele we lacked sufficient data on the fetal karyotype and time or mode of delivery. In one case, triploidy was diagnosed in a female fetus with PRUV accompanied by tetralogy of Fallot (TOF), and single umbilical artery (SUA). In this particular case, the gestation finished in a stillbirth and spontaneous delivery at 34 gestational weeks (Table 2).

**Table 2:** Summary of findings in the 12 cases with persistent right umbilical vein diagnosed during routine second or third trimester fetal scanning

Case	GA at diagnosis (weeks)	GA at delivery (weeks)	Mode of delivery	Fetal sex	Birth weight (g)	Additional findings	Karyotype
1	20 + 1	40 + 0	Caesarean section	Female	3050	Isolated	46 xx
2	24 + 5	41 + 2	Spontaneous	Female	3630	Isolated	
3	24 + 2	40 + 3	Spontaneous	Male	3850	Isolated	46 xy
4	28 + 3	39 + 3	Spontaneous	Male	3370	Isolated	
5	22 + 1	35 + 1	Caesarean section	Male	2200	Omphalocele	46 xy
6	21 + 1	36 + 2	Caesarean section	Male	2950	Isolated	
7	20 + 4	40 + 2	Caesarean section	Male	3750	Isolated	
8	19 + 6	No data	No data	No data	No data	Omphalocele	No data
9	20 + 5	34 + 3	Spontaneous Stillbirth	Female	695	TOF, SUA, IUGR	69XXX
10	20 + 4	40 + 1	Spontaneous	Female	3570	Isolated	
11	21 + 0	35 + 4	Spontaneous	Male	2570	Isolated	
12	20 + 6	41 + 2	Spontaneous	Male	4020	Isolated	

GA, gestational age

## Discussion

Clinical significance of PRUV depends on its type and concomitant malformations. These may exacerbate the prognosis, affect the management during pregnancy and mode of delivery. Congenital anomalies of the fetal precordial venous system, which include different kinds of lesions, are observed in about 1.32% of pregnancies.^1^, ^17^ The fetal venous system plays a key role in fetal circulation, as it transports oxygenated blood to the fetal heart. A significant portion of the oxygenated blood flows directly from the DV to the left atrium through the foramen ovale. The congenital absence of DV, which may coexist with PRUV, results in dysregulation and subsequent volume overload. In severe cases, cardiomegaly, polyhydramnios and fluid accumulation may occur.^11^, ^18^ PRUV is an uncommon antenatal finding and a proper diagnosis remains a challenge for many doctors. The widespread use of colour Doppler and 3D US applications have facilitated the *in utero* diagnosis of a number of abnormalities in fetal circulation, including those in the umbilical cord or the fetal portal system.^19^

Two variants of PRUV are described^10^

Type 1 – the intrahepatic PRUV (PRUV‐I) – is the most prevalent, reported in 95% of cases.^1^, ^12^ In PRUV‐I, the UV passes lateral to the right side of the gallbladder, connects to the right PV, and then bends toward the stomach. The DV is usually present and there is a little interference in haemodynamics. This type of PRUV has a good prognosis. All our cases were of the intrahepatic type, and DV was detected in all of them.

Type 2 is the extrahepatic PRUV (PRUV‐E), where the UV connects directly to the right atrium or the IVC.^1^, ^12^ PRUV‐E is associated with DV agenesis and a poorer prognosis.^3^, ^5^, ^11^, ^20^, ^21^ If the DV is absent, the blood returns directly to the heart. This might increase the haemodynamic burden. The affected fetuses suffer from volume overload and severe haemodynamic effects that result in fetal hydrops. There are several case reports of agenesis of the DV with fetal hydrops.^11^, ^22^

Typically, PRUV is an isolated anomaly^6^, ^12^ however, it may be accompanied by other disorders in the gastrointestinal tract, cardiovascular or genitourinary systems. In the study reported by Blazer *et al*., among 69 fetuses with PRUV, nine fetuses (13%) had other sonographic abnormalities but among them only one anomaly (1.4%) was clinically significant.^6^ The extrahepatic types of PRUV are more frequently associated with these anomalies.^6^ According to the analysis by Weichert *et al*., additional anomalies were present in all extrahepatic PRUV cases (*n* = 16).^5^ The prognosis is thus worse than that of PRUV‐I.^15^ Aneuploidy testing showed no chromosomal abnormalities in any of the analysed PRUV‐I cases in the study by Sun *et al*.^23^ In the systemic review by Lide *et al*.,^13^ 76.3% of 240 cases of PRUV‐I were isolated anomalies, but the rest were accompanied by heart abnormalities (7.9%), placental or umbilical cord anomalies (7%), genitourinary malformations (6.3%) or central nervous system malformations (3.8%). Genetic disorders were diagnosed in 1.3% of fetuses. Cardiovascular malformations also seem to be the leading coexisting anomaly in other reports.^15^, ^23^ The studies by Weichert *et al*. and Wolman *et al*., reported 74.4% and 76.4% respectively of isolated PRUV that had no associated anomalies, which is similar to Lide *et al*.^4^, ^5^, ^13^ In our study, PRUV‐I cases were isolated in 75%, which is consistent with the literature.^4^, ^5^, ^13^ Out of 12 PRUV cases, three (25%) had different malformations. Two of the diagnosed fetuses had omphalocele, and in one case there were multiple abnormalities (TOF, SUA, PRUV). Only two isolated and two non‐isolated PRUV patients from our study group underwent fetal karyotyping. Aneuploidy was found only in the fetus with multiple abnormalities. The authors of the other reports suggest that invasive prenatal diagnosis should be limited to the usual obstetric indications because of the low prevalence of genetic abnormalities in isolated PRUV‐I, but may be reasonable in the presence of additional abnormalities.^5^, ^13^, ^15^

According to the databases of the worldwide literature, most of the investigations into PRUV have been either of a retrospective or review nature, and not prospective. They suggest that the incidence of PRUV amounts to 0.08–0.4% of all pregnancies.^5^, ^6^, ^13^, ^15^ In the most recent systematic review of the literature covering 166 548 women, the prevalence of PRUV was found to be 0.13%.^13^ Our own data show a much higher incidence of PRUV than was traditionally considered and which is higher than in other European studies.^5^, ^15^ This higher incidence may be the result of the methodology as our study is prospective unlike the other retrospective and review investigations. The first prospective study by Wolman *et al*. (2002) based on 8950 low‐risk patients reports 17 PRUV cases (0.18%).^4^ In comparison to the reports mentioned above, in our population the incidence of PRUV was 1/196, constituting 0.5%. Our incidence of PRUV is comparable to those from the other two prospective studies carried out, varying from 0.46% in a Taiwanese report based on 1302 pregnant women to 0.49% in an Israeli cohort of 1810 low‐risk women.^16^, ^17^ These differences may result from the size of the population under study.

In conclusion, PRUV is an uncommon prenatal finding but its incidence may be higher than traditionally thought. The most frequent form of PRUV is intrahepatic type without any coexisting malformations and this may be the reason why the anomaly can be overlooked on screening US. Prenatal screening for PRUV can be easily performed in all pregnant patients. The combination of 2D with multiplanar reconstruction allows the precise identification of the location of intra‐abdominal UV, its shape and direction. A diagnosis of anomalous venous anatomy or improper function should be followed by a thorough fetal morphology scan in order to exclude any other malformations, especially those of the cardiovascular system. In isolated PRUV‐I fetal karyotyping is not necessary, but the decision should be individual and depend on other US markers of aneuploidy.

## References

[1] Yagel S , Kivilevitch Z , Cohen SM *et al* The fetal venous system, Part II: ultrasound evaluation of the fetus with congenital venous system malformation or developing circulatory compromise. Ultrasound Obstet Gynecol 2010; 36: 93–111. 20205158 10.1002/uog.7622

[2] Yagel S , Kivilevitch Z , Cohen SM *et al* The fetal venous system, Part I: normal embryology, anatomy, hemodynamics, ultrasound evaluation and Doppler investigation. Ultrasound Obstet Gynecol 2010; 35: 741–750. 20205155 10.1002/uog.7618

[3] Perez‐Cosio C , Sheiner E , Abramowicz JS . Four‐vessel umbilical cord: not always a dire prognosis. J Ultrasound Med 2008; 27: 1389–1391. 18716150 10.7863/jum.2008.27.9.1389

[4] Wolman I , Gull I , Fait G *et al* Persistent right umbilical vein: incidence and significance. Ultrasound Obstet Gynecol 2002; 19: 562–564. 12047534 10.1046/j.1469-0705.2002.00678.x

[5] Weichert J , Hartge D , Germer U *et al* Persistent right umbilical vein: a prenatal condition worth mentioning? Ultrasound Obstet Gynecol 2011; 37: 543–548. 20922781 10.1002/uog.7764

[6] Blazer S , Zimmer EZ , Bronshtein M . Persistent intrahepatic right umbilical vein in the fetus: a benign anatomic variant. Obstet Gynecol 2000; 95: 433–436. 10711558 10.1016/s0029-7844(99)00564-5

[7] Kalache K , Romero R , Goncalves LF *et al* Three‐dimensional color power imaging of the fetal hepatic circulation. Am J Obstet Gynecol 2003; 189: 1401–1406. 14634577 10.1067/s0002-9378(03)00774-9

[8] Hofstaetter C , Plath H , Hansmann M . Prenatal diagnosis of abnormalities of the fetal venous system. Ultrasound Obstet Gynecol 2000; 15: 231–241. 10846780 10.1046/j.1469-0705.2000.00066.x

[9] Kaczmarek P , Borowski D , Wegrzyn P *et al* Clinical significance of the doppler evaluation in ductus venosus, hepatic veins and pulmonary veins. Ginekol Pol 2005; 76: 498–504. 16149270

[10] Jeanty P . Persistent right umbilical vein: an ominous prenatal finding? Radiology 1990; 177: 735–738. 2243979 10.1148/radiology.177.3.2243979

[11] Achiron R , Hegesh J , Yagel S *et al* Abnormalities of the fetal central veins and umbilico‐portal system: prenatal ultrasonographic diagnosis and proposed classification. Ultrasound Obstet Gynecol 2000; 16: 539–548. 11169348 10.1046/j.1469-0705.2000.00220.x

[12] Chaoui R , Kalache KD , Hartung J . Application of three‐dimensional power Doppler ultrasound in prenatal diagnosis. Ultrasound Obstet Gynecol 2001; 17: 22–29. 11244651 10.1046/j.1469-0705.2001.00305.x

[13] Lide B , Lindsley W , Foster MJ *et al* Intrahepatic persistent right umbilical vein and associated outcomes: a systematic review of the literature. J Ultrasound Med 2016; 35: 1–5. 10.7863/ultra.15.01008 26635256

[14] Hajdu J , Marton T , Kozsurek M *et al* Prenatal diagnosis of abnormal course of umbilical vein and absent ductus venosus–report of three cases. Fetal Diagn Ther 2008; 23: 136–139. 18046072 10.1159/000111594

[15] Martinez R , Gamez F , Bravo C *et al* Perinatal outcome after ultrasound prenatal diagnosis of persistent right umbilical vein. Eur J Obstet Gynecol Reprod Biol 2013; 168: 36–39. 23317918 10.1016/j.ejogrb.2012.12.019

[16] Yang PY , Wu JL , Yeh GP *et al* Prenatal diagnosis of persistent right umbilical vein using three‐dimensional sonography with power Doppler. Taiwan J Obstet Gynecol 2007; 46: 43–46. 17389188 10.1016/S1028-4559(08)60105-9

[17] Yagel S , Cohen SM , Valsky DV *et al* Systematic examination of the fetal abdominal precordial veins: a cohort study. Ultrasound Obstet Gynecol 2015; 45: 578–583. 24919785 10.1002/uog.13444

[18] Hofstaetter C , Gudmundsson S . Venous Doppler in the evaluation of fetal hydrops. Obstet Gynecol Int 2010; 2010: 430157. 20454533 10.1155/2010/430157 PMC2864890

[19] Kivilevitch Z , Gindes L , Deutsch H , Achiron R . In‐utero evaluation of the fetal umbilical‐portal venous system: two‐ and three‐dimensional ultrasonic study. Ultrasound Obstet Gynecol 2009; 34: 634–642. 19953568 10.1002/uog.7459

[20] Bradley E , Kean L , Twining P , James D . Persistent right umbilical vein in a fetus with Noonan's syndrome: a case report. Ultrasound Obstet Gynecol 2001; 17: 76–78. 11244662 10.1046/j.1469-0705.2001.00243.x

[21] Baschat AA . Ductus venosus Doppler for fetal surveillance in high‐risk pregnancies. Clin Obstet Gynecol 2010; 53: 858–868. 21048453 10.1097/GRF.0b013e3181fbb06d

[22] Corbacioglu A , Aslan H , Dagdeviren H , Ceylan Y . Prenatal diagnosis of abnormal course of umbilical vein and ductus venosus agenesis: report of three cases. J Clin Ultrasound 2012; 40: 590–593. 22362124 10.1002/jcu.21883

[23] Sun L , Wang Y . Demographic and perinatal outcome data of fetuses with SUA/PRUV. J Matern Fetal Neonatal Med 2017; 3: 1–6. 10.1080/14767058.2017.1309384 28320222

